# Opportunities for human resources for health and rehabilitation: a response to Jesus et al.

**DOI:** 10.1186/s12960-017-0244-x

**Published:** 2017-09-30

**Authors:** Jessica Power, Joanne McVeigh, Brynne Gilmore, Malcolm MacLachlan

**Affiliations:** 1 Centre for Global Health, Trinity College Dublin, 7-9 Leinster Street South, Dublin 2, Ireland; 2 Department of Psychology, Maynooth University, John Hume Building, North Campus, Maynooth, Co. Kildare, Ireland; 3 Centre for Rehabilitation Studies, Faculty of Medicine and Health Sciences, Stellenbosch University, P.O. Box 241, Cape Town, 8000, South Africa; 4 Olomouc University Social Health Institute, Palacký University, Univerzitní 22, 771 11 Olomouc, Czech Republic

**Keywords:** Rehabilitation, Workforce, “Global Health”

## Abstract

We welcome Jesus et al.’s paper, which makes an important contribution to the under-researched area of the physical rehabilitation workforce. The authors present recommendations to “advance a policy and research agenda for ensuring that an adequate rehabilitation workforce can meet the current and future rehabilitation health needs” (p. 1). We argue that their perspective could however be strengthened by adopting a stronger global perspective, including consideration of the needs of low-resource settings. In particular, we highlight the integral role of more effective sector and inter-sectoral governance, the opportunity to support the development of community-based rehabilitation (CBR), the lessons that can be learnt from human resources for health (HRH) research and practice more generally, and the recent developments in the global provision of assistive technologies. Each of these issues has important implications and contributions to make to advance the policy and research agenda for the global rehabilitation workforce.

We welcome Jesus et al.’s paper [1], which makes an important contribution to the under-researched area of the physical rehabilitation workforce. The authors present recommendations to “advance a policy and research agenda for ensuring that an adequate rehabilitation workforce can meet the current and future rehabilitation health needs” (p. 1). We argue that their perspective could however be strengthened by adopting a stronger global perspective, including consideration of the needs of low-resource settings. We wish to complement their original article by highlighting further key areas. In particular, we highlight the integral role of more effective sector and inter-sectoral governance, the opportunity to support the development of community-based rehabilitation (CBR), the lessons that can be learnt from human resources for health (HRH) research and practice more generally, and the recent developments in the global provision of assistive technologies. Each of these issues has important implications and contributions to make to advance the policy and research agenda for the global rehabilitation workforce.

Jesus et al. propose a policy agenda, highlighting the need to “adapt policy options to different contexts (e.g. rural vs urban), even within a country” (p. 1), and provide recommendations for both local and global policymakers. However, the six rehabilitation workforce challenges outlined by the paper do not sufficiently recognise the integral role of governance and leadership in addressing these, including strategies to increase the priority of rehabilitation on the health agenda. Dieleman and Hilhorst [2] specify that the influence of governance is undervalued in addressing the HRH crisis, at both country and global levels, including issues of accountability, planning, implementation, monitoring, corruption and transparency. Governance impacts on all other health system functions and can lead to improved performance of a health system, including effective delivery of the interface between rehabilitative and general health services [3, 4]. Health workforce challenges necessitate strengthening health governance in addition to human resource systems, to generate change in the overall health system [5]. The Joint Learning Initiative [6] asserts that “workforce development should be seen as a political-technical process, shaped by history, bureaucratic procedures, labour markets, and political accommodations of diverse interests … leadership is thus crucial to strengthen national ownership of workforce strategies” (pp. 66-67). There is an opportunity to build on global guidance documents, for example the Joint Learning Initiative [6], the World Health Organization’s (WHO) Global Code of Practice on the International Recruitment of Health Personnel [7] and WHO Global Strategy 2030 [8], providing potential solutions to challenges faced in rehabilitation workforce planning. Although we note that Jesus et al. cited these references in their article, we believe that they have more potential to address rehabilitation workforce challenges than was developed in their paper.

With the rapidly growing ageing population, and the majority of persons with disabilities living in low- and middle-income countries (LMICs), there is a need to consider the global context when addressing rehabilitation workforce challenges [9, 10]. Jesus et al. suggest six rehab-workforce challenges for the century. However, they make little mention of CBR, the strategy favoured by the WHO [11] and civil society organisations in resource-poor settings [12]. CBR has been steadily growing since the 1970s, so that it is now present in an estimated 90 countries [9]. The CBR matrix, which provides guidance on components within CBR, has health and rehabilitation as key elements within this. We believe that Jesus et al. underemphasise the opportunity and role of CBR in addressing many of the weaknesses and threats that the authors identify, including coverage gaps, barriers, lack of physically accessible sites, international migration and access outside of hospital settings (see for instance [13-18]).

While Jesus et al. focus on setting an agenda for physical rehabilitation, it is important to consider that often professionals working with people with physical disabilities may need to have a multi-faceted skillset [19]. This can often include softer skills outside of traditional technical skills [20–22]. For example, within CBR, professionals often have to take on an advocacy role to reduce stigma associated with disability [9]. While Jesus et al. allude to negative societal beliefs as a barrier to access, they do not discuss rehabilitation professionals’ potential role in addressing this through advocacy at a policy level, or community-level interventions to change attitudes. In our experience, rehabilitation professionals who work in remote areas often need to be able to provide services to not just those with physical disabilities, but also to those experiencing difficulties with cognition, activities of daily living (ADLs), and communication etc., which may be outside of their original physical rehabilitation training skillset [23]. Such skillsets may therefore need to be expanded or alternatively “shifted”.

While the role of task-shifting in providing services was alluded to by Jesus et al., this approach deserves much more attention. The evidence for task-shifting in rehabilitation is slowly growing [24]. However, we can look to other areas of health where task-shifting has been shown to be an effective strategy, such as for maternal and child health [25, 26] and HIV/AIDS interventions [27], or well-documented workforce considerations, such as training, motivation, supervision and retention [28–30]. Learning from challenges, opportunities and evidence-based examples from these disciplines, specifically in relation to task-shifting and providing services for vulnerable populations, is a viable strategy for the rehabilitation workforce.

One of the most significant global initiatives in the field of physical rehabilitation is WHO’s GATE (Global Cooperation on Assistive Technology) programme, which seeks to produce a step-change in the provision and use of affordable quality assistive technology (AT) [31]. Following a three-round Delphi procedure with 200 participants and a global survey completed by over 10,000 respondents from 161 countries, the Priority Assistive Products List (APL) was agreed at a Consensus Conference in Geneva in 2016 [32]. The strategic development of this list focuses on four thematic areas — products, policies, provision and personnel. The latter, personnel, is likely to involve both up-skilling existing rehabilitation professions and developing new cohorts, using task-shifting, to ensure that AT expertise is available at the community level in some of the world’s most resource-poor contexts. This then should also be a key factor in planning for the human resources necessary to support AT users in the rehabilitation sector.

Jesus et al. draw attention to the important and under-researched area of physical rehabilitation health workforce challenges. It is important that while setting a global agenda, such an effort should encompass the challenges and potential solutions for high-, medium- and low-resource settings, as well as urban and rural settings. We have sought to complement Jesus et al.’s important article by highlighting some of the ways in which this can be done.

## Abbreviations

ADLs: Activities of daily living
APL: Priority Assistive Products List
AT: Assistive technology
CBR: Community-based rehabilitation
GATE: Global Cooperation on Assistive Technology
HRH: Human resources for health
LMICs: Low- and middle-income countries
WHO: World Health Organization
